# Synergistic Drug-Induced Diffuse Alveolar Hemorrhage in a Polysubstance User: A Case of Inhaled Cocaine and Fentanyl Toxicity

**DOI:** 10.7759/cureus.87582

**Published:** 2025-07-09

**Authors:** Belissa A Lopez-Pena, Natalia Canevaro-Lugo, Angel U Davila-Cardona, Maniel Ruiz-Ramos, Maria C Perez-Mitchell, Ricardo Fernandez-Gonzalez

**Affiliations:** 1 Internal Medicine, San Juan City Hospital, San Juan, USA; 2 Pulmonary and Critical Care Medicine, San Juan City Hospital, San Juan, USA

**Keywords:** acute respiratory failure, addiction medicine, bronchoalveolar lavage, cocaine inhalation, critical care, diffuse alveolar hemorrhage (dah), drug-induced lung injury, fentanyl toxicity, polysubstance abuse, pulmonary hemorrhage

## Abstract

Diffuse alveolar hemorrhage (DAH) is a rare but life-threatening pulmonary condition characterized by bleeding into the alveolar spaces, most commonly associated with autoimmune or vasculitic disorders. However, drug-induced DAH, particularly from the inhalation of substances like cocaine and fentanyl, is an emerging concern. A 37-year-old homeless male with a history of intravenous and inhaled polysubstance abuse was found unresponsive and treated with intranasal naloxone for presumed opioid overdose. Upon arrival, he exhibited respiratory distress, pink frothy sputum, severe hypoxemia, and acute renal failure with refractory hyperkalemia. Imaging revealed diffuse bilateral alveolar infiltrates, and large volumes of bright red blood were suctioned during intubation. Bronchoalveolar lavage confirmed DAH with numerous hemosiderin-laden macrophages. Infectious and autoimmune workups were negative, while urine toxicology was positive for both fentanyl and cocaine. Management included lung-protective mechanical ventilation, high-dose corticosteroids, and continuous renal replacement therapy, with clinical improvement and successful extubation after ten days. This case underscores the importance of considering drug-induced DAH in patients with acute respiratory failure and polysubstance use, even in the absence of hemoptysis.

## Introduction

Diffuse alveolar hemorrhage (DAH) is a life-threatening pulmonary syndrome resulting from bleeding into the alveolar spaces, manifesting clinically with hemoptysis, hypoxemic respiratory failure, and bilateral infiltrates on imaging. Although most frequently associated with autoimmune vasculitides, DAH is increasingly recognized in the context of drug-induced lung injury. Inhaled substances, particularly cocaine, are well-established contributors to pulmonary capillaritis due to their vasoconstrictive and pro-inflammatory properties [1-3]. More recently, fentanyl, an ultra-potent synthetic opioid, has emerged as a critical player in the pathogenesis of acute lung injury and has been increasingly implicated in overdose-related deaths across the United States, particularly among vulnerable and polysubstance-using populations [1-4]. The concurrent use of fentanyl and cocaine, often encountered in street drug mixtures, presents a dangerous synergy capable of precipitating catastrophic pulmonary outcomes [5]. We report the case of a 37-year-old man with polysubstance abuse who developed DAH and acute renal failure following fentanyl and cocaine inhalation, and we provide a detailed discussion of the pathophysiologic mechanisms, clinical management, and broader public health implications.

## Case presentation

A 37-year-old homeless male with a known history of intravenous and inhaled fentanyl and cocaine use was found unresponsive in a public location. Emergency medical services administered intranasal naloxone for presumed opioid overdose, after which the patient regained consciousness and complained of worsening dyspnea and pleuritic chest discomfort. Upon arrival at the emergency department, he appeared acutely ill, with a respiratory rate of 32 breaths per minute, SpO₂ of 82% on a non-rebreather mask, heart rate of 118 bpm, and blood pressure of 142/85 mmHg. Physical examination revealed bilateral coarse crackles, frothy pink sputum, and 2+ lower extremity edema.

**Figure 1:**
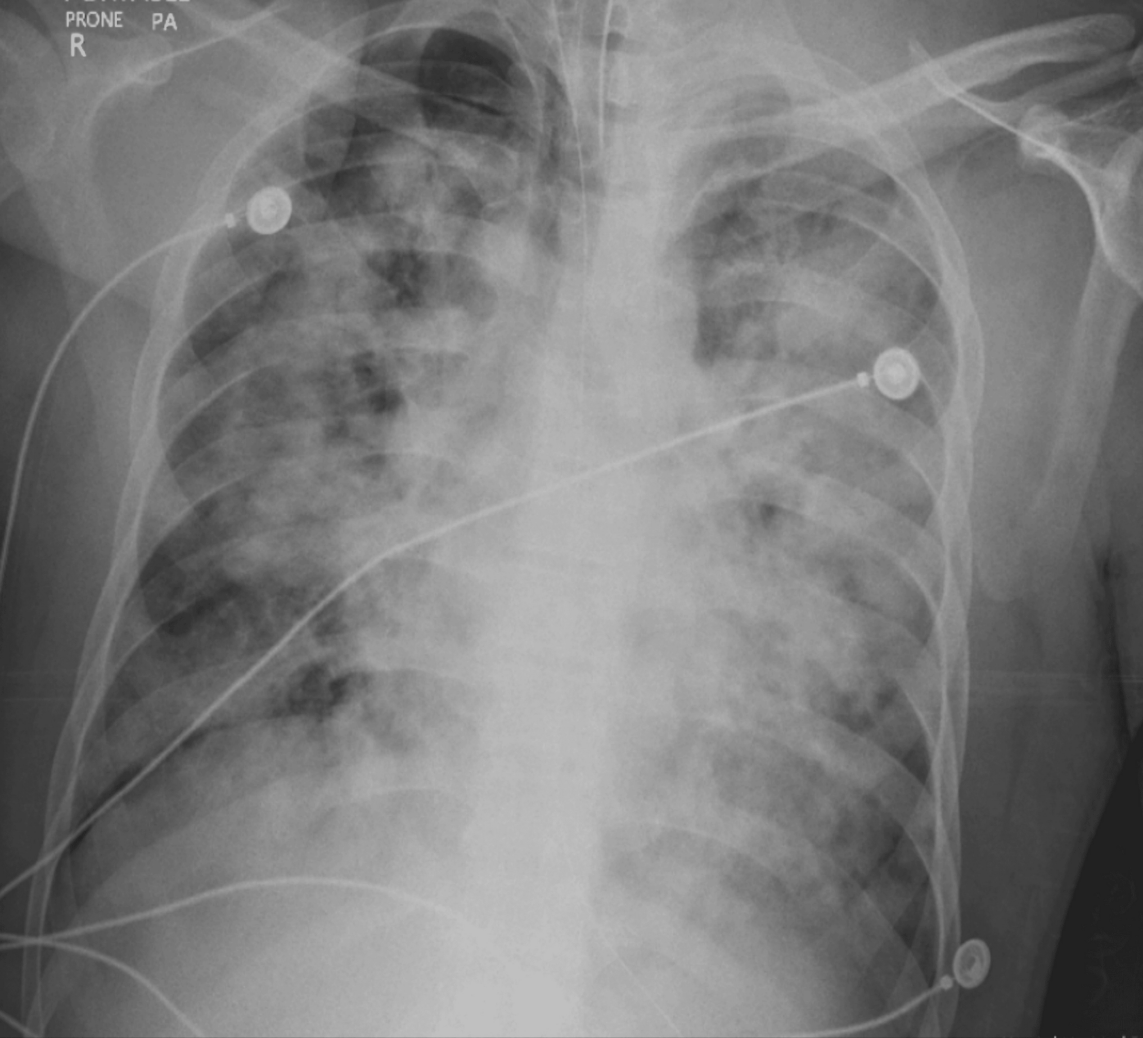
Chest X-ray showing diffuse bilateral alveolar infiltrates characterized by extensive patchy and confluent opacities.

Initial laboratory evaluation revealed acute renal dysfunction with blood urea nitrogen (BUN) of 42 mg/dL and creatinine of 3.2 mg/dL. He had severe hyperkalemia of 8.2 mEq/L, which was refractory to insulin-dextrose, calcium gluconate, and potassium-binding resins. Arterial blood gas showed acidemia with a pH of 7.21, PaCO₂ 52 mmHg, and PaO₂ 58 mmHg. Chest radiograph demonstrated diffuse bilateral alveolar infiltrates (Figure 1). Due to progressive respiratory failure, the patient was intubated. During the procedure, copious amounts of bright red, frothy blood were suctioned from the airway, raising high suspicion for DAH.

**Figure 2:**
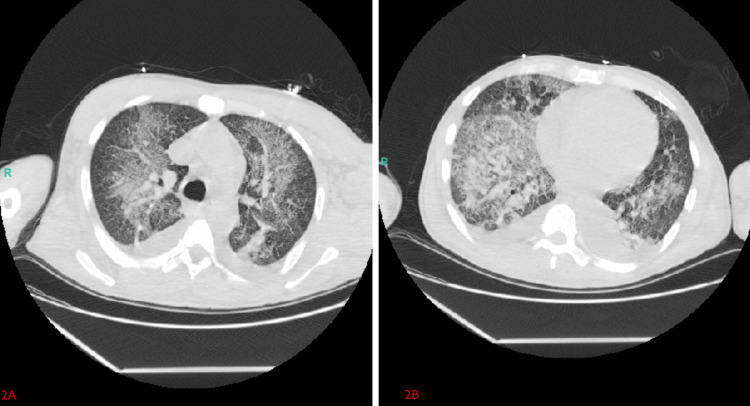
Axial CT chest imaging. A: axial CT image at the mid-lung level demonstrates extensive bilateral multifocal and multilobar ground-glass opacities with superimposed interlobular septal thickening; B: axial CT image at the lung bases reveals confluent opacities in the bilateral lower lobes along with small-to-moderate bilateral pleural effusions.

**Figure 3:**
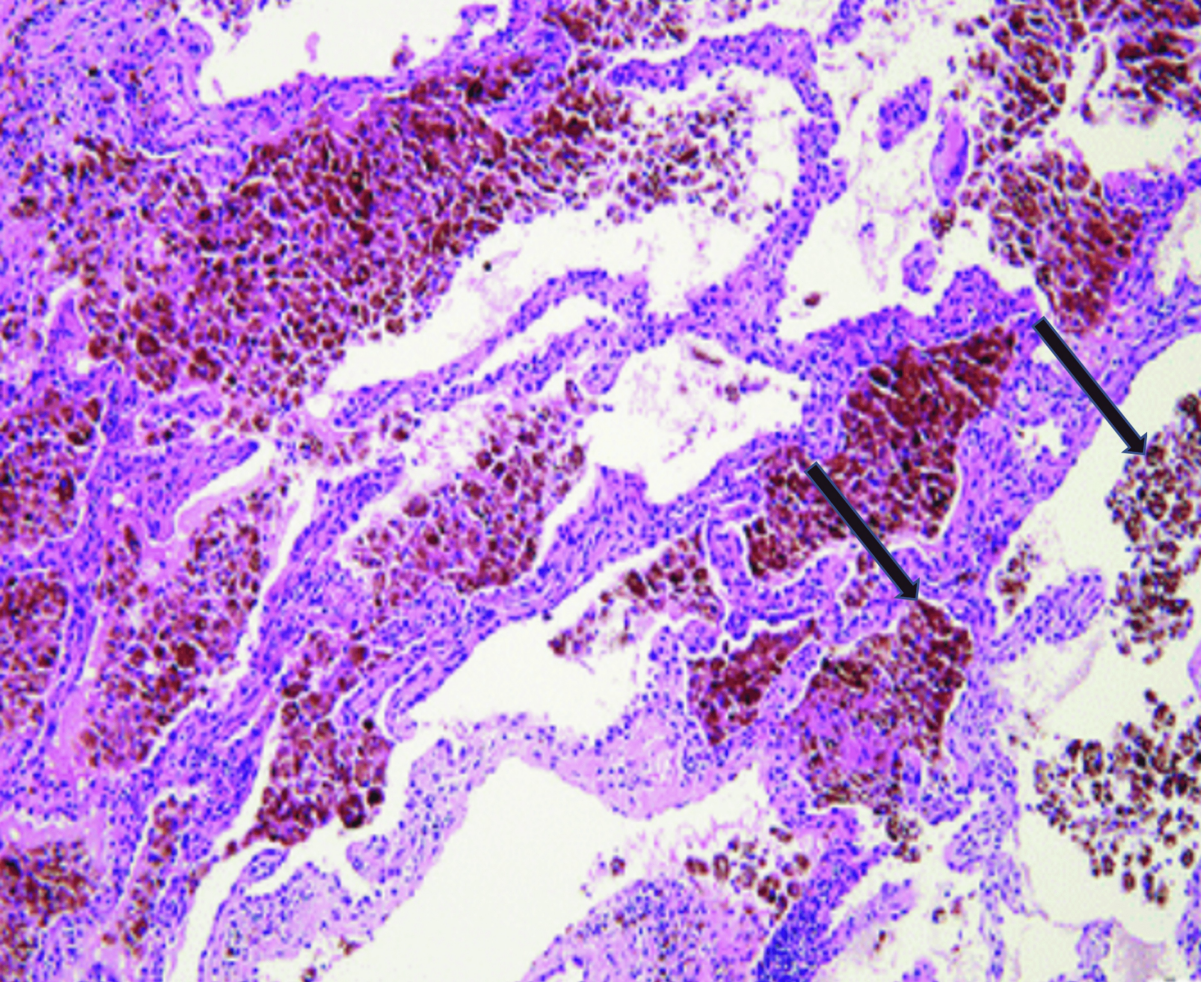
Histologic from BAL demonstrates numerous hemosiderin-laden macrophages within alveolar spaces. BAL: bronchoalveolar lavage

A CT scan of the chest revealed extensive bilateral multifocal and multilobar ground-glass opacities with superimposed interlobular septal thickening and confluent opacities in the lower lobes, along with small-to-moderate bilateral pleural effusions (Figure 2). He was admitted to the intensive care unit, where bronchoscopy with bronchoalveolar lavage (BAL) showed progressively bloody aliquots, and Prussian blue staining confirmed the presence of hemosiderin-laden macrophages, consistent with alveolar hemorrhage (Figure 3). An infectious workup, including blood and respiratory cultures, viral panels, and testing for atypical pathogens, was negative. Autoimmune testing, including antinuclear antibody (ANA), antineutrophil cytoplasmic antibody (ANCA), and anti-glomerular basement membrane (anti-GBM) antibodies, was also negative. Urine toxicology confirmed the presence of fentanyl and cocaine.

**Figure 4:**
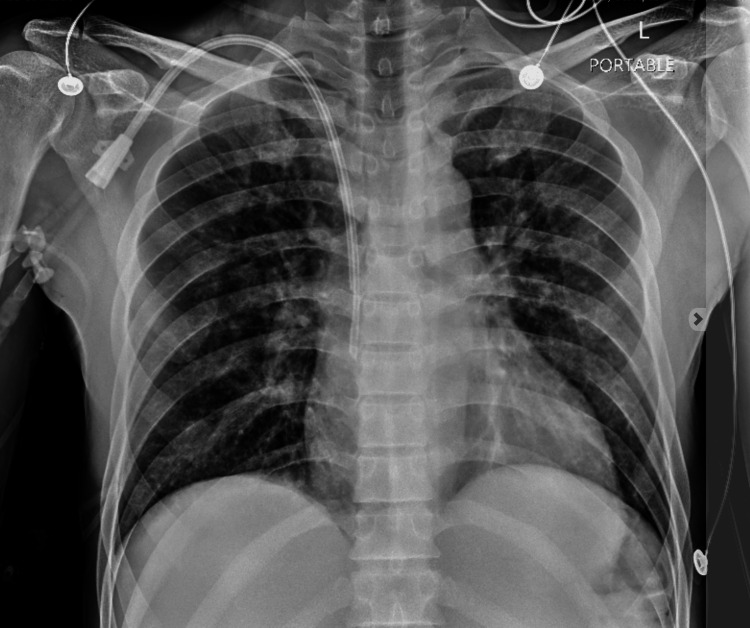
A chest X-ray after corticosteroid treatment shows a right internal jugular (IJ) central line with the tip at the cavoatrial junction, stable interstitial thickening, and no effusion, consolidation, or pneumothorax.

Management included mechanical ventilation with lung-protective strategies, high-dose corticosteroids (methylprednisolone 1 g IV daily for three days), and continuous renal replacement therapy for metabolic derangements and volume control. Given persistent hypoxemia, the patient was placed in the prone position during the acute phase to optimize oxygenation. He gradually improved and was extubated after ten days of intensive care (Figure 4). He was discharged with referrals to pulmonology and addiction medicine.

## Discussion

This case illustrates a rare but increasingly relevant manifestation of drug-induced lung injury: DAH precipitated by the inhalation of cocaine and fentanyl [1-3]. The pathogenesis of DAH in polysubstance use involves multiple overlapping mechanisms. Cocaine-induced vasospasm and small-vessel vasculitis are well-documented causes of alveolar capillary rupture [2,4]. The drug stimulates the sympathetic nervous system, generating oxidative stress and endothelial injury, which increases vascular permeability and promotes hemorrhage. Fentanyl, with its potent central respiratory depressant effects, induces hypoventilation, hypercapnia, and acidosis, all of which exacerbate pulmonary capillary stress [5,6]. Its role in increasing pulmonary hydrostatic pressure may contribute to non-cardiogenic edema, further weakening the alveolar-capillary barrier [1].

Inhaled fentanyl is an emerging route of abuse increasingly associated with acute pulmonary complications. Unlike intravenous administration, smoking or vaporizing fentanyl introduces high concentrations of the drug and adulterants directly into the alveolar space, bypassing hepatic metabolism and delivering cytotoxic effects to the alveolar epithelium. Proposed mechanisms of injury include oxidative stress, surfactant disruption, and direct alveolar membrane toxicity, as well as particulate-induced inflammation from cutting agents such as talc and silica [5-7]. These factors may predispose to DAH, especially in the context of stimulant co-use. Recent public health data indicate that fentanyl-related overdose deaths have surged over the past five years, with increasing cases linked to inhalational use in vulnerable populations, including those experiencing homelessness [6,8,9].

Literature suggests that when these two drugs are co-administered, often unknowingly, they result in synergistic effects that amplify endothelial disruption [1,5]. Cocaine-induced vasoconstriction combined with fentanyl-induced hypoventilation creates a hemodynamic milieu that predisposes to alveolar capillary rupture. Moreover, the administration of naloxone, while life-saving, rapidly reverses opioid-induced sedation, provoking a catecholamine surge that can transiently elevate pulmonary vascular pressures and worsen alveolar bleeding [10]. This phenomenon may be particularly harmful in individuals with underlying endothelial injury from vasotoxic substances.

Compounding this pulmonary insult are systemic metabolic derangements, such as the patient’s acute kidney injury and refractory hyperkalemia. Uremia contributes to capillary fragility and impairs platelet function, while hyperkalemia exacerbates acidosis and impairs oxygen delivery [6]. These factors collectively destabilize pulmonary homeostasis, reinforcing the multifactorial pathogenesis of DAH in this population.

Supportive care remains the cornerstone of management. In this case, the patient was managed with lung-protective ventilation, targeting tidal volumes of 6-8 mL/kg of ideal body weight and maintaining plateau pressures under 30 cm H₂O. High positive end-expiratory pressure (PEEP) was employed to prevent alveolar collapse and minimize further bleeding. The patient was also placed in the prone position for several sessions during the acute phase to improve oxygenation and redistribute perfusion, consistent with best practices in severe hypoxemic respiratory failure [11]. Fluid management was carefully balanced to avoid volume overload, with renal replacement therapy initiated early to correct hyperkalemia, acidosis, and fluid retention. Corticosteroids were administered despite limited evidence for their use in drug-induced DAH; their rationale lies in mitigating inflammation and capillary leak, extrapolated from autoimmune-related DAH literature [12]. Other therapies, such as recombinant activated factor VII, have been reported in severe cases, although their use remains controversial and was not indicated in this patient.

Long-term management must address both pulmonary and behavioral health outcomes. Pulmonary follow-up with repeat imaging and pulmonary function testing is warranted to assess for residual fibrosis or chronic lung disease. Equally important is the integration of addiction medicine services, as relapse prevention and substance use rehabilitation are essential to reducing recurrence. This case also serves as a reminder of the disproportionate burden borne by vulnerable populations. Homelessness, lack of access to healthcare, and untreated psychiatric illness often converge in individuals with polysubstance abuse, increasing their risk of critical illness and poor outcomes [6].

The broader context of the opioid epidemic, particularly the rise in fentanyl-laced street drugs, underscores the urgency for clinician awareness. The mixing of potent opioids with stimulants like cocaine has created a new spectrum of clinical presentations that challenge traditional diagnostic frameworks [13]. This evolving landscape calls for greater investment in harm-reduction strategies, including drug-checking services, supervised consumption facilities, and education on the dangers of fentanyl adulteration. Finally, further research is urgently needed. Longitudinal studies should assess the long-term pulmonary sequelae of drug-induced DAH, while clinical trials should evaluate the efficacy of corticosteroids and adjunctive therapies in this setting. Public health research must also focus on interventions targeting at-risk populations, addressing the root causes of addiction, and structural barriers to care [13].

## Conclusions

Drug-induced DAH is an underrecognized but deadly consequence of the inhalation of cocaine and fentanyl, especially when used concurrently. This case exemplifies the complex interplay of vasotoxicity, respiratory depression, systemic metabolic dysfunction, and social vulnerability in producing severe pulmonary injury. Clinicians should maintain a high index of suspicion for DAH in patients presenting with acute respiratory failure and a history of polysubstance use, even in the absence of hemoptysis. Early bronchoscopy, lung-protective mechanical ventilation, and supportive therapies are key to survival. As fentanyl continues to dominate the illicit drug landscape, a multidisciplinary response spanning critical care, addiction medicine, and public health will be necessary to mitigate the growing threat of substance-induced lung injury.
